# Early Reoperations after Primary Repair of Jejunoileal Atresia in Newborns

**DOI:** 10.21699/jns.v5i4.444

**Published:** 2016-10-10

**Authors:** Fanny Yeung, Yuk Him Tam, Yuen Shan Wong, Siu Yan Tsui, Hei Yi Wong, Kristine Kit Yi Pang, Christopher H Houben, Jennifer Wai Cheung Mou, Kin Wai Chan, Kim Hung Lee

**Affiliations:** Division of Paediatric Surgery and Paediatric Urology, Department of Surgery, Prince of Wales Hospital, The Chinese University of Hong Kong, Hong Kong, China

**Keywords:** Jejunoileal atresia, Primary resection and anastomosis, Reoperation

## Abstract

**Aim:** To review nine-year experience in managing jejuno-ileal atresia (JIA) by primary resection and anastomosis and identify factors associated with reoperations.

**Methods:** From April 2006 to May 2015, all consecutive neonates who underwent bowel resection and primary anastomosis for JIA were analyzed retrospectively. Patients with temporary enterostomy were excluded. Patient demographics, types of atresia, surgical techniques, need for reoperations, and long-term outcomes were investigated.

**Results:** A total of forty-three neonates were included, in which nineteen (44.2%) of them were preterm and fourteen (32.6%) were of low birth weight. Thirteen patients (30.2%) had jejunal atresia whereas thirty patients (69.8%) had ileal atresia. Volvulus, intussusception and meconium peritonitis were noted in 12, 8, and13 patients, respectively. Eight patients (18.6%) had short bowel syndrome after operation. Ten patients (23.3%) required reoperations from 18 days to 4 months after the initial surgery due to anastomotic stricture (n=1), adhesive intestinal obstruction (n=1), small bowel perforation (n=2) and functional obstruction (n=6). Prematurity and low birth weight were associated with functional obstruction leading to reoperation (p=0.04 and 0.01 respectively). The overall long-term survival was 97.7%. All surviving patients achieved enteral autonomy and catch-up growth at a median follow-up of 4.7 years.

**Conclusion:** Long-term survival of JIA after primary resection and anastomosis are excellent. However, patients have substantial risk of early reoperations to tackle intraabdominal complications.

## INTRODUCTION

Jejunoileal atresia (JIA) is a major cause of neonatal intestinal obstruction, with an incidence ranging from 1.3 to 2.9 per 10,000 live births[1-3].With advancement of neonatal intensive care, anesthesia, parenteral nutrition and operative techniques, the survival figure was reported to be around 80% in 1990s [4] and further climbed to more than 90% in the 21st century [1,5].

The operative management depends on the site of the atresia, the specific anatomical findings, associated gastrointestinal tract anomalies and the length of the remaining small bowel. In contemporary practice, resection of the proximally dilated bowel along with the atretic segment and primary anastomosis is the recommended choice of operation whenever possible [4,5,6]. The frequency of temporary stoma has decreased from over 20% in the 1970s to 10% in the 1990s [6]. A temporary enterostomy, however, is necessary in less favorable conditions with doubtful vascular integrity of bowel, significant intraperitoneal contamination by meconium peritonitis, and excessive discrepancy in the diameter between the distal and the proximally dilated bowel which is too long to be resected or tapered [4,5,7,8].

Despite significant decline in mortality, infants with JIA experience substantial morbidity after primary surgery [5]. Abdominal reoperation rate varied from 14% to 25% due to postoperative complications [6,7,9]. There are few studies, however, that investigated risk factors associated with re-laparotomy following primary repair of JIA. In this study, we reviewed our experience in managing patients with congenital JIA over a 9-year period and analyzed the long-term outcomes after proximal bowel resection with primary anastomosis and the factors associated with reoperation.

## MATERIALS AND METHODS

After obtaining the approval of the clinical research ethics committee of our institution, we conducted a retrospective analysis of all consecutive neonates who underwent primary bowel resection and anastomosis for JIA between April 2006 and May 2015 in our hospital which is a tertiary referral center. 

Data about gestation, birth weight, associated anomalies, antenatal diagnosis, type of JIA, possible etiology, surgical techniques and postoperative outcomes were analyzed. JIA was classified into type I to IV as described by Grosfeld et al [10]. Short bowel syndrome(SBS) was defined by residual small bowel length less than 50cm, 75cm and 100cm for premature newborns of gestation between 28 and 36 weeks, term newborns, and older children respectively [5,8]. Prematurity and low-birth-weight (LBW) were defined by gestation of less than 37 weeks at birth and birth weight of less than 2.5kg respectively. Reoperation was defined by any re-laparotomy for complications related to previous primary repair of the JIA.

Categorical data were reported in percentage as frequency. Continuous variables were expressed as median with interquartile range (IQR). Continuous and categorical data were compared by Mann-Whitney U and chi-square tests, respectively. Statistical significance was defined by p value less than 0.05.

## RESULTS

A total of 49 neonates including 28 boys and 21 girls with operation for JIA were identified. Forty-three patients (87.8%) who had primary repair by resection of proximal atretic segment with primary anastomosis were included in the study whereas six patients who underwent enterostomy were excluded. The median age at operation was 1.0 day (IQR 1.0-3.0)

Among the 43 patients with primary anastomosis, 19 patients (44.2%) were premature. Median birth weight was 2.87 kg (IQR 2.03-3.30) and 14 patients (32.6%) had LBW. One patient had associated congenital anomaly with small ventricular septal defect. Antenatal ultrasonography was suggestive of intestinal obstruction in 19 patients (44.2%) and polyhydramnios was noted in 3 patients (7.0%). 

Upon laparotomy, thirteen patients (30.2%) had jejunal atresia whereas thirty patients (69.8%) had ileal atresia. Nine patients (20.9%) had type I atresia, seven patients (16.3%) had type II atresia, nineteen patients (44.2%) had type III atresia and eight patients (18.6%) had type IV multiple atresias. Volvulus and intussusception were identified in 12 and 8 patients respectively, and were believed to be the etiology for JIA. The findings of volvulus and intussusception, usually evident on the distal atretic segments, were consistent with the generally accepted belief that JIA develops after intrauterine mesenteric vascular insults leading to bowel resorption and formation of atresia [4,5]. Meconium peritonitis was present in 13 patients and reflected the occurrence of intrauterine bowel perforations. Meconium peritonitis shares the same etiology as JIA formation and its presence in some of our patients was considered to be the consequence rather than the cause of JIA [5]. 

The median small bowel length remaining after proximal bowel resection and primary anastomosis was 101.5cm (IQR 73.3-110.0). Eight patients (18.6%) had SBS. The discrepancy in diameter between the proximal and the distal bowel at anastomosis was less than or equal to 3 to 1 ratio. Thirty-three patients (76.7%) recovered uneventfully from the primary surgery and achieved full enteral feeding at a median of 22 days after the initial surgery, ranging from 10 to 96 days.

Ten patients (23.3%) required abdominal reoperation at a median time interval of 34.5 days, ranging from 18 days to 4 months from the first operation. Reoperation was not associated with prematurity (p=0.67), LBW (p=0.18), jejunal/ileal atresia (p=0.84), in-utero intussusception (p=0.43), in-utero volvulus (p=0.53), meconium peritonitis (p=0.42) and SBS (p=0.30). The indications of reoperation were anastomotic stricture (n=1), adhesive intestinal obstruction complicated with small bowel volvulus (n=1), small bowel perforation (n=2), and functional obstruction (n=6).

The patient with anastomotic stricture had resection of the stricture and bowel re-anastomosis at the reoperation. For the patient with volvulus due to adhesion band, small bowel resection and primary anastomosis was performed. The two patients with bowel perforations presented with sudden onset of distended abdomen with pneumoperitoneum on 23 and 34 days after the first operation. One of them was found to have a 2cm perforation on the anti-mesenteric side of the ileum 4cm distal to the previous anastomosis. The previous anastomosis was resected en bloc with the perforated bowel and re-anastomosis was performed. The initial pathology of this patient was type IIIb atresia with apple-peel configuration of the distal small bowel. The other patient had a small punctate perforation at 25cm from the previous anastomosis and simple repair of the perforation was performed.

On the other hand, six patients had reoperation for functional obstruction, in which five of them were born preterm at median gestation of 31 weeks and had LBW with median weight of 1.6 kg. Both prematurity (p=0.04) and LBW (p=0.01) were associated with functional obstruction leading to re-laparotomy. These patients experienced persistent intestinal obstruction after the bowel resection and primary anastomosis. They presented with feeding intolerance, recurrent bouts of abdominal distension with dilated proximal bowel loops on abdominal radiograph when feeding was increased, and sepsis from bacterial overgrowth in dilated bowel loops. Contrast follow-through study was performed and showed patent anastomosis with transition from grossly dilated bowel on the proximal side to collapsed bowel on distal side of the anastomosis. Repeat laparotomy was performed at a median of 37 days, ranging from 18 days to 3 months after the first operation in view of persistent intestinal obstruction. Upon reoperation, patency of the previous anastomosis was confirmed in all 6 patients after adhesiolysis and bowel just proximal to the anastomosis was most dilated while bowel distal to anastomosis was collapsed. In 5 patients, the previous anastomosis was resected together with further proximal bowel resection. Immediate bowel re-anastomosis was performed in 3 patients while temporary stomas were fashioned in 2 patients. Histological examination of the resected anastomosis confirmed patency without fibrotic stricture and confirmed presence of ganglion cells in all 5 patients. The remaining one patient was treated by a side-to-side anastomosis between the dilated and collapsed bowel without resecting the original anastomosis as the primary anastomotic site was close to duodeno-jejunal flexure. For the 2 patients with temporary stomas, one recovered uneventfully after closure of stoma at her 3 months of age. The other one had recurrent functional obstruction after closure of the stoma and required another operation of side-to-side anastomosis between the dilated and collapsed bowel at his age of 4 months. His enteral feeding tolerance improved afterwards although full enteral feeding was unable to be achieved because of recurrent bowel distension. This patient, however, died at the age of 9 months because of sepsis and parenteral nutrition-related liver failure, and was the only mortality in the whole study group.

Except for the mortality case, the other 5 patients who underwent reoperation for functional obstruction eventually achieved full enteral autonomy from 24 -84 days after the reoperation. At a median follow-up of 4.7 years, all the surviving 42 patients achieved enteral autonomy and showed normal growth. The overall long-term survival was 97.7%. Mortality was associated with functional obstruction requiring reoperation (p=0.01), but not with prematurity (p=0.26), LBW (p=0.15), types of atresia (p=0.17), meconium peritonitis (p=0.51), abdominal reoperation (0.07) or SBS (0.63).

## DISCUSSION

Almost 90% of our patients born with JIA were amenable to primary repair without the need of temporary enterostomy. However, almost one-fourth of our patients needed an abdominal reoperation for postoperative complications after primary repair for JIA. The risk of reoperation is substantial and our finding appears to be in agreement with the impression in the existing literature although this issue has not been widely investigated. A previous study reported that 15 of 60 patients (25%) with JIA required a second abdominal operation for complications [9]. Another study recruiting 83 patients reported that 14.5% required repeat laparotomy for adhesive intestinal obstruction and another 6% for anastomotic leakage or stricture [6].

It has been known for several decades that the dilated proximal bowel should not be used to reconstruct the continuity of the gastrointestinal tract in instances of JIA as functional obstruction will result from the failure of the dilated bowel wall to generate adequate intraluminal pressure for effective peristalsis despite a patent anastomosis [11-13]. The improved neonatal care with total parenteral nutrition, and the recommended surgical principle of liberal resection of the dilated proximal bowel with primary anastomosis whenever the remaining bowel length permits, have contributed to the improved long-term survival of newborns with JIA to 84% and the decline of temporary enterostomy to 10% in the 1990s[4,6]. It is, however, a clinical and subjective judgement by the operating surgeon to strike a balance between resecting adequate proximally dilated bowel to prevent leaving behind dysmotile bowel which would result in functional obstruction, and preserving as much bowel as possible to minimize the effect of short bowel syndrome. 

We have followed the recommendation in performing primary anastomosis that the discrepancy in diameter between the two ends of the bowel after proximal resection should not be excessive and the ratio should be less than 1 to 5 [5]. Nevertheless, we still encountered 6 cases (14%) of persistent functional obstruction after proximal resection and primary anastomosis and required reoperation. All these 6 patients did not tolerate enteral feeding at the expected pace after the initial surgery while the proximal bowel became progressively dilated and complicated with bacterial overgrowth and sepsis. Contrast follow-through study unequivocally showed patent anastomosis. Although peritoneal adhesions were found expectedly during re-laparotomy for these patients, the transition from distended to collapsed bowel was consistently at the previous anastomosis and the resected anastomosis did not show any evidence of stricture. Adhesiolysis alone does not suffice to correct functional obstruction and further proximal resection of the dysmotile bowel is necessary if the remaining bowel length permits. From our findings, prematurity and LBW were significantly associated with more functional obstruction requiring reoperation. We hypothesize that the immature intestinal motility explains this observation. Small intestinal motor activity has been found to be present at 16 weeks of gestation, but coordinated contraction and peristalsis is not established until 36 weeks [14-16]. Prematurity and LBW had also been previously shown to be significant predictors of increased mortality of JIA by Walker et al, with term infants having a 98% survival rate compared with 87% for preterm infants[1,9].

The incidence of functional obstruction requiring reoperation after primary anastomosis for JIA has not been well reported. A study in Netherlands reported that 13% of patients developed small bowel obstruction which occurred as early as within a month after the initial surgery, but the authors did not specify the cause of the obstruction nor did they report the management [5]. Kumaran et al reported 11% of prolonged adynamic ileus [6] while Piper et al reported 8% of their patients required reoperation of tapering enteroplasty [9] which presumably was indicated for the retained dilated bowel with dysmotility although the authors did not elaborate further. Notably, previous studies reported a relatively high incidence rates from 7 to 24% of adhesive intestinal obstruction requiring repeat laparotomy as early as within a month after the primary operation [4,6,7,9]. We speculate that some of the reported cases of adhesive intestinal obstruction, particularly those who had early reoperation, could be related to functional obstruction. The progressive dilatation of the dysmotile bowel on the proximal side of the anastomosis creates a funnel-shaped configuration and could result in more kinking and adhesions near the anastomotic site.

In our study, there was only one patient who required reoperation due to purely adhesive intestinal obstruction. The patient had been discharged from hospital with full enteral feeding and readmitted for acute intestinal obstruction. Laparotomy found adhesion bands causing volvulus of a segment of small bowel distant from the initial anastomosis. On the other hand, two patients had isolated bowel perforations that occurred 3 to 5 weeks after the primary surgery for JIA in which the etiology of perforations was uncertain. Unnoticed injury to bowel wall or its blood supply during dissection in the initial surgery could result in delayed bowel perforation. This is possible particularly in one of the cases which the blood supply to the distal bowel with apple-peel configuration was precarious. 

Other findings in the present study are in agreement with the existing literature. Prenatal diagnosis of intestinal obstruction was 44.2% in our study, which was comparable to 41% in a population-based study in Japan [2]. Like previous studies [4,5,17], we found in-utero volvulus and intussusception in 28% and 19% of our patients respectively as the etiological factors for JIA formation. After proximal bowel resection and primary anastomosis, 18% of our patients had SBS and the figure was comparable to the 15% reported in one of the largest case series among 114 neonates with JIA [5].The authors suggested higher morbidity and mortality rates among patients with SBS who were dependent on prolonged parenteral nutrition [5]. From our findings, mortality was not associated with SBS but with functional obstruction. However, the only mortality case in the present study resulted from sepsis with parenteral nutrition-related liver failure. This patient died before our introduction of fish oil-based lipid preparation in parenteral nutrition which carries a lower risk to liver injury [18]. Total parenteral nutrition was required in 47 to 79% of neonates after operation for JIA, and dependency was significantly associated with the length of remaining small bowel [5-7]. Improved parenteral nutrition with less cholestasis would further decrease the morbidity and mortality after surgery for JIA.

Our study was limited by its retrospective nature and the study period spanned a decade which there had been continuous improvement in neonatal intensive care with better parenteral nutrition being introduced. The operations were performed by a group of attending surgeons with variations in their experience and seniority. Nevertheless, our study provides updated data on the long-term outcomes after primary repair for JIA. We have reaffirmed the findings that vast majority of neonates born with JIA can be managed by primary repair without enterostomy. In contemporary neonatal care setting, the long-term survival after primary anastomosis can reach over 95%. However, parents should be alerted to the substantial risk of early reoperations to tackle intraabdominal complications during the informed consenting process. Despite various technical advancements in neonatal care, it remains to be a subjective judgement of the operating surgeon on how much proximally dilated bowel to be resected. It can be challenging even to experienced surgeons to strike a balance between preserving as much bowel as possible and removing adequately the bowel expected to have dysmotility.

## Footnotes

**Source of Support:** None

**Conflict of Interest:** None
